# Optimization of dye adsorption time and film thickness for efficient ZnO dye-sensitized solar cells with high at-rest stability

**DOI:** 10.1186/1556-276X-7-688

**Published:** 2012-12-28

**Authors:** Wei-Chen Chang, Chia-Hua Lee, Wan-Chin Yu, Chun-Min Lin

**Affiliations:** 1 Institute of Organic and Polymeric Materials, National Taipei University of Technology, Taipei, 10608, Taiwan; 2 Green Energy and Environment Research Laboratories, Industrial Technology Research Institute, Hsinchu, 31053, Taiwan

**Keywords:** Zinc oxide, Dye-sensitized solar cells, Dye adsorption time, Film thickness, Conversion efficiency, At-rest stability, Electrochemical impedance spectroscopy

## Abstract

Photoelectrodes for dye-sensitized solar cells were fabricated using commercially available zinc oxide (ZnO) nanoparticles and sensitized with the dye N719. This study systematically investigates the effects of two fabrication factors: the ZnO film thickness and the dye adsorption time. Results show that these two fabrication factors must be optimized simultaneously to obtain efficient ZnO/N719-based cells. Different film thicknesses require different dye adsorption times for optimal cell performance. This is because a prolonged dye adsorption time leads to a significant deterioration in cell performance. This is contrary to what is normally observed for titanium dioxide-based cells. The highest overall power conversion efficiency obtained in this study was 5.61%, which was achieved by 26-μm-thick photoelectrodes sensitized in a dye solution for 2 h. In addition, the best-performing cell demonstrated remarkable at-rest stability despite the use of a liquid electrolyte. Approximately 70% of the initial efficiency remained after more than 1 year of room-temperature storage in the dark. To better understand how dye adsorption time affects electron transport properties, this study also investigated cells based on 26-μm-thick films using electrochemical impedance spectroscopy (EIS). The EIS results show good agreement with the measured device performance parameters.

## Background

Dye-sensitized solar cells (DSSCs) are regarded as promising low-cost solar cells with high light-to-energy conversion efficiency. Systems based on titanium dioxide (TiO_2_) nanoparticle films sensitized with ruthenium (Ru)-based dyes have achieved a light-to-energy conversion efficiency of more than 11% [1,2]. Other metal oxides, including tin dioxide, indium (III) oxide, niobium pentoxide, and zinc oxide (ZnO), have also been used as photoelectrode materials [3-5]. Among these materials, ZnO has attracted considerable attention because it has an energy-band structure similar to that of TiO_2_ but possesses a higher electron mobility and allows more flexibility in synthesis and morphologies [6,7].

The photovoltaic performance of a DSSC relies on the characteristics of its photoanode, which plays a central role in converting light into electrical energy. A DSSC photoanode typically consists of a mesoporous oxide film on a transparent conducting glass substrate. Dye molecules that capture photons from light during device operation are attached to the surface of oxide film. Photoexcitation of the dye molecules leads to the injection of electrons into the oxide film. Therefore, an oxide film with a large interfacial surface area and superior electron transport properties is vital for strong light harvesting and efficient device performance. Consequently, numerous researchers have attempted to develop novel nanostructures with these desirable properties [8-12]. Another important strategy that has been widely adopted in DSSCs to boost optical absorption is light scattering [13]. The basic principle of the light scattering method is to confine light propagation and extend the traveling distance of light within the oxide film. In this way, the opportunity of photon absorption by the dye molecules is increased, so is the cell conversion efficiency. In traditional DSSCs, the porous photoelectrode typically consists of nanocrystallites of approximately 20 nm in diameter to ensure a large interfacial surface area; to generate light scattering, submicron-sized particles are incorporated into the nanocrystalline film. These submicron-sized light scatterers can either be mixed into the nanocrystalline film [14,15] or form a scattering layer on the top of the nanocrystalline film [16-20]. In addition to submicron-sized particles, some other nanostructures, such as nanowires [21-23] and nanotubes [24,25] have also been studied as light scatterers in DSCCs. Recently, a promising three-dimensional nanostructure that has been developed to fulfill multiple functions in DSSCs is nanocrystallite aggregates [26-29]. These aggregates not only provide a large interfacial surface area, but also generate light scattering because they are composed of nanoparticles that assemble into submicron aggregates. Employing nanocrystallite aggregates can avoid the drawbacks of using large particles as light scatterers in conventional DSSCs. Mixing the large particles into the nanocrystalline film unavoidably causes a decrease in the interfacial surface area of the film, whereas placing the large particles on top of the nanocrystalline film brings about a limited increase in the interfacial surface area of the film.

Regardless of the film nanoarchitecture employed, film thickness and dye adsorption time are two important factors that must be considered during photoanode fabrication. Increasing the total interfacial surface area of the porous film by raising the film thickness is simple, which boosts the amount of dye adsorbed and, thus, light absorption. Thus, raising the film thickness can increase the short-current density (*J*_SC_) [21,30]. However, a thick film also aggravates unwanted charge recombination and poses more restrictions on mass transfer. Consequently, both the open-current voltage (*V*_OC_) and overall conversion efficiency decline [14,21,30,31]. Therefore, film thickness must be optimized to obtain efficient cells.

Another key fabrication factor is the dye adsorption time, which determines the quantity and the nature of the adsorbed dye molecules. The dye adsorption time should be sufficiently long so that the interfacial surface of the oxide film is completely covered with a monolayer of dye molecules. In fabricating TiO_2_-based photoanodes, the length of the dye adsorption time is first determined and then applied to all film thicknesses during the subsequent thickness optimization process [32-34]. This is because TiO_2_ is insensitive to prolonged sensitization times because of its higher chemical stability. Conversely, a prolonged dye adsorption time in ZnO-based photoanodes often significantly deteriorates cell performance. Thus, varying film thicknesses may require different dye adsorption times for optimal cell performance. Compared to TiO_2_, ZnO is less stable with acidic dyes, such as Ru-based N3 and N719 dyes. The formation of Zn^2+^/dye aggregates is a result of ZnO dissolution in these acidic dye solutions [32,35-37]. The formation of dye aggregates has also been reported for indoline dyes [38]. Ideally, the oxide surface should be covered with a monolayer of dye molecules to achieve efficient electron injection. When dye molecules undergo aggregation, electron injection becomes less efficient, and overall conversion efficiency declines. However, Yan et al. [39], on the other hand, observe the surface etching of ZnO nanoflowers after a long sensitization time. Surface etching also leads to a significant loss in overall conversion efficiency. For ZnO-based cells, it is essential to optimize the dye adsorption time to minimize the formation of dye aggregates and the damage to ZnO surfaces. Because the dye molecules must penetrate the mesoporous oxide film before they attach to the interfacial surface, the optimal dye adsorption time likely depends on the thickness of the ZnO film. Thus, this study investigates both the film thickness and the dye adsorption time. Although these two factors have been individually investigated before and certain studies have reported the influences of dye concentration and adsorption time on DSSC performance [32,36], a detailed and systemic study of the effects of film thickness and dye adsorption time for ZnO-based DSSCs is lacking.

This study reports the preparation of DSSC photoelectrodes using commercially available ZnO nanoparticles sensitized with the acidic N719 dye. This study also systematically investigates the influences of ZnO film thickness and dye adsorption time on the performance of the resulting DSSCs. To further understand the effect of dye adsorption time, electrochemical impedance spectroscopy (EIS) was used to investigate the electron transport characteristics of the fabricated cells. This study shows the correlation between *J*_SC_ and dye loading as a function of the dye adsorption time and reports the at-rest stability of the best-performing cell.

## Methods

### Fabrication of solar cells

ZnO films (active area 0.28 cm^2^) of various thicknesses (14 to 35 μm) were deposited on fluorine-doped tin oxide (FTO) substrates (8 to 10 Ω/□, 3 mm in thickness, Nippon Sheet Glass Co. Ltd, Tokyo, Japan) by screen printing. Screen-printable ZnO paste was prepared by dispersing commercially available ZnO nanoparticles (UniRegion Bio-Tech, Taiwan) in an equal proportion of α-terpineol (Fluka, Sigma-Aldrich, St. Louis, MO, USA) and ethyl cellulose. Before dye adsorption, the ZnO films were sintered at 400°C for 1 h to remove any organic material in the paste. This thermal treatment sintered the nanoparticles together to form an interconnecting network. Dye sensitization was achieved by immersing the sintered ZnO films in a 0.5 mM solution of *cis*-diisothiocyanato-bis(2,2^′^-bipyridyl-4,4^′^-dicarboxylato)-ruthenium(II) bis(tetrabutylammonium) (N719, Solaronix; Solaronix SA, Aubonne, Switzerland). The solvent used to prepare the dye solution consisted of equal parts of acetonitrile and *tert*-butanol. Dye sensitization was performed at room temperature, and the adsorption time varied from 0.5 to 4.5 h. The electrodes loaded with the N719 dye were then washed with acetonitrile and dried in air. Platinum (Pt)-coated FTO glass (Nippon Sheet Glass, 8–10 Ω/□, 3 mm in thickness) served as the counter electrode, which was prepared by placing a drop of H_2_PtCl_6_ solution on an FTO glass and subsequently sintering the glass at 400°C for 20 min. The ZnO photoanode and the counter electrode were sealed together with a 60-μm-thick hot-melting spacer (Surlyn, DuPont, Wilmington, DE, USA), and the inner space was filled with a volatile electrolyte. The electrolyte was composed of 0.1 M lithium iodide, 0.6 M 1,2-dimethyl-3-propylimid-azolium iodide (PMII, Merk Ltd., Taipei, Taiwan), 0.05 M I_2_ (Sigma-Aldrich), and 0.5 M *tert*-butylpyridine (Sigma-Aldrich) in acetonitrile.

### Characterization

The morphologies of the ZnO nanoparticle films were examined by field-emission scanning electron microscopy (FE-SEM; Nova230, FEI Co., Hillsboro, OR, USA). The crystalline phases of the ZnO films were determined by X-ray diffraction (XRD) using a diffractometer (X'Pert PRO, PANalytical B.V., Almelo, The Netherlands) with Cu Kα radiation. The thickness of the ZnO nanoparticle film was measured using a microfigure-measuring instrument (Surfcorder ET3000, Kosaka Laboratory Ltd., Tokyo, Japan). Dye loading of the photoelectrode was estimated by desorbing the dye in a 10 mM NaOH aqueous solution and then measuring the absorbance of the solution using UV–vis spectroscopy (V-570, Jasco Inc., Easton, MD, USA). Photovoltaic characterization was performed under a white light source (YSS-100A, Yamashita Denso Company, Tokyo, Japan) with an irradiance of 100 mW cm^−2^ at an equivalent air mass (AM) of 1.5 on the surface of the solar cell. The irradiance of the simulated light was calibrated using a silicon photodiode (BS-520, Bunko Keiki Co., Ltd, Tokyo, Japan). Current–voltage (*J*-*V*) curves were recorded with a PGSTAT 30 potentiostat/galvanostat (Autolab, Eco-Chemie, Utrecht, The Netherlands). The evolution of the electron transport process in the cell was investigated using EIS, and the impedance measurements were preformed under AM 1.5 G illumination. The applied DC bias voltage and AC amplitude were set at open circuit voltage (*V*_OC_) of the cell and 10 mV between the working and the counter electrodes, respectively. The frequency range extended from 10^−2^ to 10^5^ Hz. The electrochemical impedance spectra were recorded using an electrochemical analyzer (Autolab PGSTAT30, Eco-Chemie) and analyzed using Z-view software with the aid of an equivalent circuit.

## Results and discussion

### Characteristics of ZnO films

**Figure 1:**
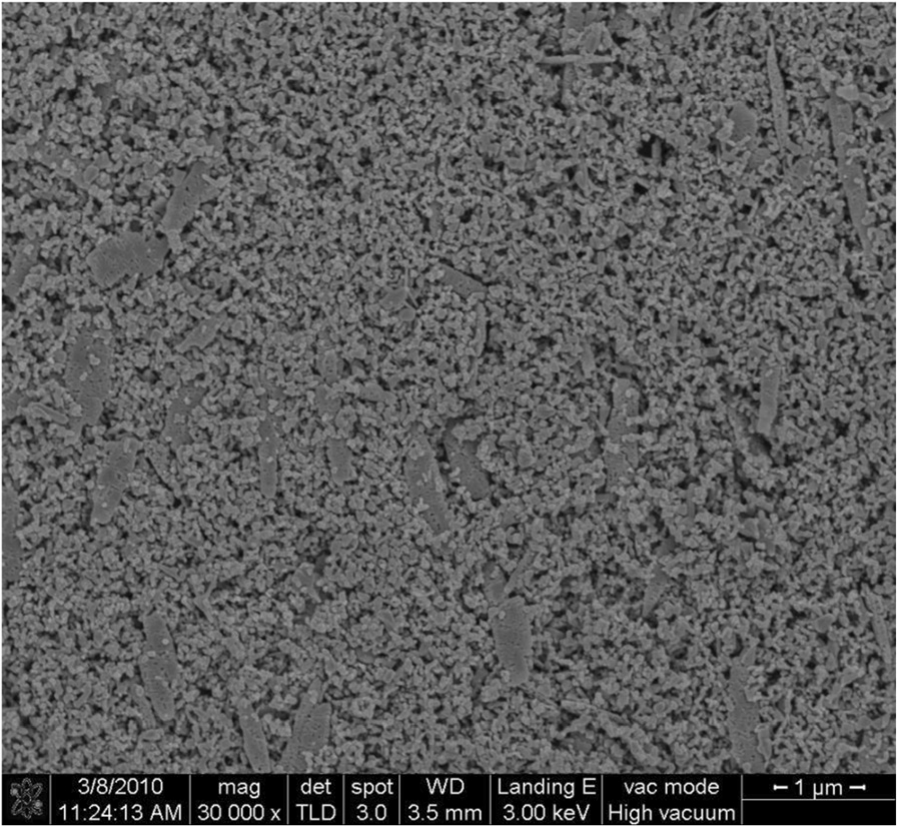
Typical FE-SEM image of sintered ZnO film on FTO substrate.

Mesoporous films composed of commercial ZnO nanoparticles were prepared by screen printing. The as-printed films were sintered at 400°C for 1 h before dye sensitization to remove organic materials in the screen-printing paste. The FE-SEM image in Figure 1 provides a typical top view of the sintered ZnO film, which is uniform and highly porous. This figure also shows that the ZnO particles in the film have two sizes: most are approximately 20 nm in diameter, whereas some are rod-shaped and have an average dimension of 200 × 500 nm. Because of their sizes, these rod-shaped particles can serve as light scatterers in the visible region of incident light, enhancing light harvesting in the resulting device [14,15,22].

**Figure 2:**
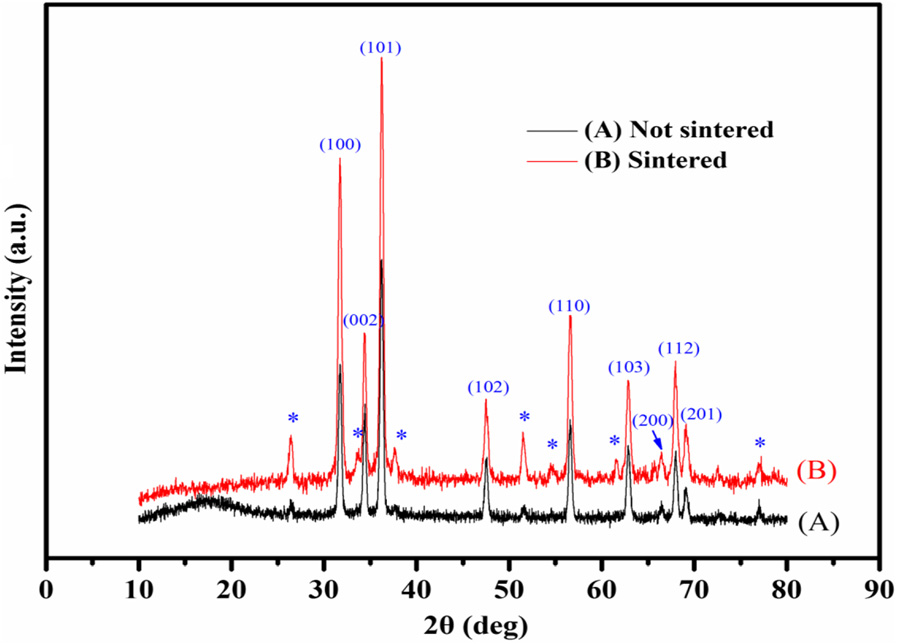
**XRD patterns of ZnO films.** (A) Not sintered and (B) sintered at 400°C for 1 h. The asterisk denotes the FTO substrate.

Figure 2 shows XRD patterns of the ZnO films before and after sintering. These two samples exhibited similar patterns except for differences in the peak intensity. Apart from those corresponding to the FTO substrate, the diffraction peaks can be indexed to the hexagonal wurtzite ZnO (JCPDS card no. 79–0206). No other diffraction peaks were found in both cases, indicating that the prepared ZnO films are of the pure wurtzite phase, and no phase transformation occurs during thermal treatment. The diffraction peaks of the ZnO film became shaper after sintering, implying that the thermal treatment raised the crystallinity of the ZnO film. Based on the XRD data, average crystallite size was estimated using the Scherrer's equation:

(1) $$D=\frac{0.89\lambda }{B\cos\theta },$$

where 0.89 is the Debye-Scherrer's constant, *λ* is the X-ray wavelength (0.15406 nm), *θ* is the Bragg's angle (measured in radians) at which the peak is observed, and *B* is the full width at half maximum. The crystallite sizes before and after sintering, as estimated from major reflections, were both approximately 20 nm. The results show that sintering did not have a significant effect on crystallite size. The estimated crystallite size matched the size of the nanoparticles in the film.

### Photovoltaic characteristics of fabricated DSSCs

**Figure 3:**
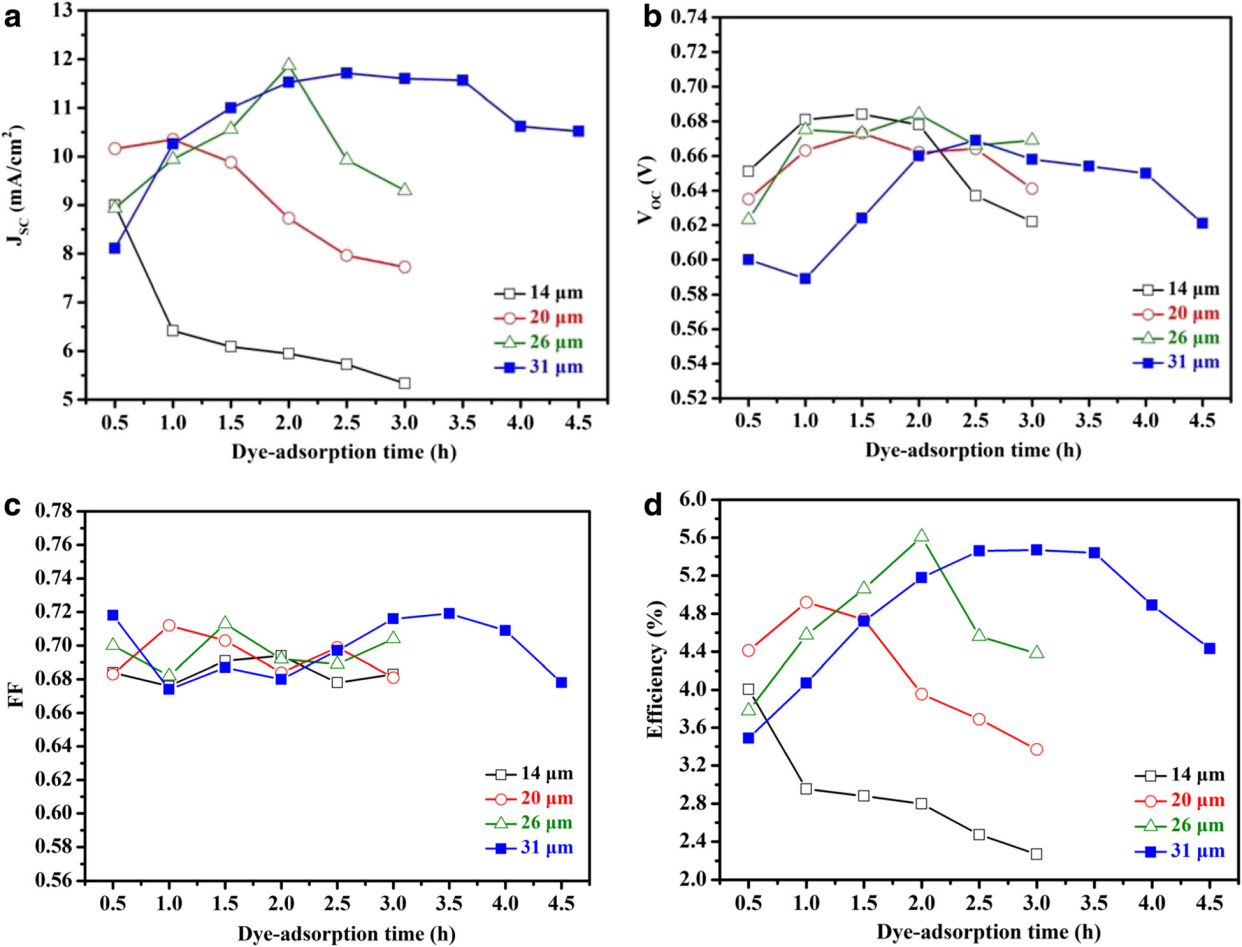
**Dependence of photovoltaic parameters of fabricated cells on dye adsorption time and ZnO film thickness.** (**a**) *J*_SC_, (**b**) *V*_OC_, (**c**) FF, and (**d**) conversion efficiency.

The performance of the fabricated DSSCs was measured under 1 sun AM 1.5 G simulated light. Figure 3 shows the dependence of various photovoltaic parameters on the dye adsorption time and the film thickness: *J*_SC_, *V*_OC_, fill factor (FF), and overall conversion efficiency. Figure 3a shows a plot of *J*_SC_ versus the dye adsorption time for various film thicknesses. Except for the thinnest photoanode (14 μm), where the *J*_SC_ values decrease continuously with increasing dye adsorption time, the *J*_SC_ values of the remaining cells exhibit a similar trend with the dye adsorption time: the *J*_SC_ values first increase as the dye adsorption time increases, reach a peak value, and then decrease as the dye adsorption time increases. The initial rise in the *J*_SC_ values with increasing dye adsorption time is likely the result of increasing dye molecule adsorption on the ZnO film. However, when the dye adsorption time becomes too long, dye molecules can aggregate on the metal oxide surface, reducing *J*_SC_[32,35-37]. Dye molecules exhibit slower electron injection or self-quench if they undergo aggregation, which can occur either before or during dye adsorption on metal oxides [40]. Figure 3a also shows that different film thicknesses require different dye adsorption times to achieve their respective peak *J*_SC_ values. The dye adsorption time required to achieve the maximum *J*_SC_ value increased from 1 h for the 20-μm photoelectrode to approximately 3 h for the 31-μm photoelectrode. The 26-μm photoelectrode achieved the highest *J*_SC_.

Figure 3b presents a comparison of *V*_OC_ values of the fabricated devices. This figure shows that the *V*_OC_ values first increase with the dye adsorption time. After reaching a maximum *V*_OC_ value, a further increase in the adsorption time leads to a decline in the *V*_OC_ value. Similar to the *J*_SC_ plot, the adsorption time required to achieve the respective maximum *V*_OC_ increases as the film thickness increases. Figure 3b also shows that the maximum *V*_OC_ values decrease slightly as the film thickness increases. This is likely the result of increased charge recombination and more restricted mass transfer with thick films. As the film thickness increases, electrons encounter a longer transport distance and recombine more easily with I_3_^−^. This results in a stronger electron transfer resistance and a shorter electron lifetime in the ZnO film [31]. The FF values shown in Figure 3c exhibit no clear trends. The FF values vary between 0.67 and 0.72, which are relatively high compared to those reported for ZnO-based DSSCs [37,41].

**Figure 4:**
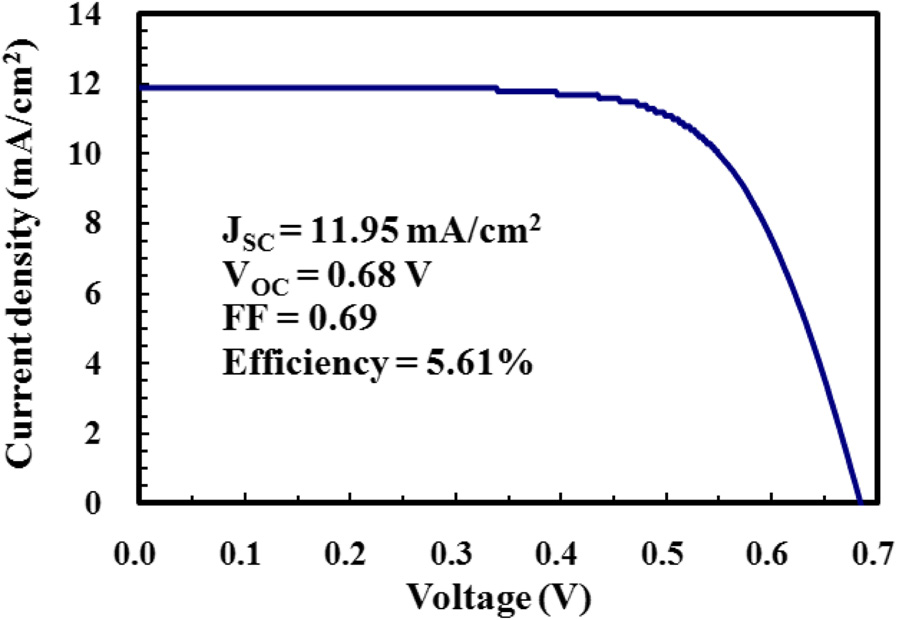
***J***-***V*curve of the best**-**performing cell.** The cell was prepared with a 26-μm film sensitized in a dye solution for 2 h.

Based on these parameters, the overall conversion efficiencies at various dye adsorption times and film thicknesses were calculated. The efficiency plot (Figure 3d) closely resembles the *J*_SC_ plot (Figure 3a). Their trends are similar and their peak values appear at the same dye adsorption times. *J*_SC_ is the efficiency-determining parameter because the dye adsorption time has a considerably stronger effect on *J*_SC_ than on other photovoltaic parameters. Figure 3d also shows that each film thickness has a unique optimal dye adsorption time at which the maximum conversion efficiency occurs. The optimal dye adsorption time determined at a given film thickness does not apply to other thicknesses. This is because the dye adsorption time is either too short or too long for other film thicknesses, resulting in considerably lower efficiencies. For example, when a dye adsorption time of 3 h (optimal for the 31-μm film) was applied to the 20-μm film, the conversion efficiency dropped from the peak value of 4.95% to approximately 3.4%, representing a 31% drop. Prolonged dye adsorption times cause dye aggregation [32,35-38] and etching of the ZnO surface [39], both of which result in performance deterioration in ZnO-based DSSCs. Conversely, TiO_2_-based DSSCs are typically less sensitive to prolonged sensitization times because of the higher chemical stability of TiO_2_[32-34]. For example, Lee et al. [33] reported similar dye loadings and conversion efficiencies for TiO_2_/N719-based DSSCs when the dye soaking time was extended from 2 to 24 h. Table 1 presents a summary of the photovoltaic characteristics of the best-performing cell for each film thickness, along with the corresponding optimal dye adsorption time. The optimal dye adsorption time varies with the film thickness; thicker films require longer dye adsorption times. In addition, the attainable conversion efficiency depends on the photoanode thickness. A photoanode that is too thin or too thick results in a lower conversion efficiency. This is because insufficient film thickness leads to a low interfacial surface area, whereas an overly thick film aggravates unwanted charge recombination and poses more restriction on mass transfer [14,21,30,31]. Consequently, for the fabrication of ZnO/N719-based DSSCs, the dye adsorption time must be optimized simultaneously with the film thickness. A 26-μm-thick photoanode soaked in the dye solution for 2 h achieved the highest conversion efficiency (5.61%) of all the cells prepared in this study. Figure 4 shows the *JV* curve of the best-performing cell measured under 1 sun AM 1.5 G simulated light.

**Table 1:** **Optimal dye adsorption times and photovoltaic characteristics of best**-**performing cell at each film thickness**

**Film thickness** (μ**m**)	**Optimal dye adsorption time** (**h**)	**Conversion efficiency** (%)	**Short**-**circuit photocurrent density** (**mA**/**cm**^**2**^)	**Open circuit voltage** (**V**)	**Fill factor**
14	0.5	3.98	9.00	0.65	0.68
20	1	4.92	10.35	0.66	0.72
26	2	5.61	11.95	0.68	0.69
31	3	5.47	11.60	0.66	0.72

**Figure 5:**
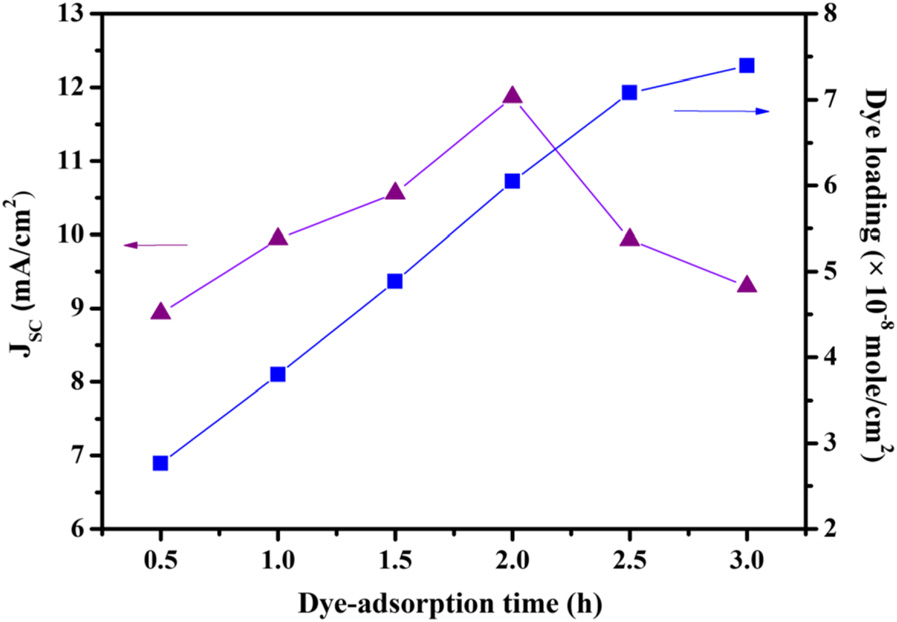
**Relationship between*J***_**SC **_**and dye loading as a function of dye adsorption time.** ZnO film thickness is 26 μm.

To better understand the effects of dye adsorption time on cell performance, this study also investigates dye loading in cells based on 26-μm-thick films. Figure 5 shows the correlation between *J*_SC_ and dye loading as a function of dye adsorption time. The amount of adsorbed dye molecules increases continuously as the adsorption time increases, whereas the *J*_SC_ value reaches a maximum value and then decreases as the dye adsorption time increases. This observation is in contrast to that reported for TiO_2_-based DSSCs, where dye loading reached saturation after 2 h of sensitization and remained at the same level even when the sensitization time increased to 24 h [33]. The continuous increase of dye loading with sensitization time observed here suggests that the *J*_SC_ deterioration is the result of dye aggregation. In this study, the ZnO film was sensitized with the weak acidic N719 dye, which was adsorbed onto the surface of ZnO particles through the carboxylic acid anchoring group. Compared to TiO_2_, ZnO is less stable in acidic dyes. Thus, immersing ZnO in an acidic dye solution for a long period can lead to ZnO dissolution and the formation of Zn^2+^/dye aggregates [32,35-37]. When dye aggregation occurs, dye molecules exhibit slower electron injection and charge recombination aggravates, resulting in lower *J*_SC_ values [36,37].

**Figure 6:**
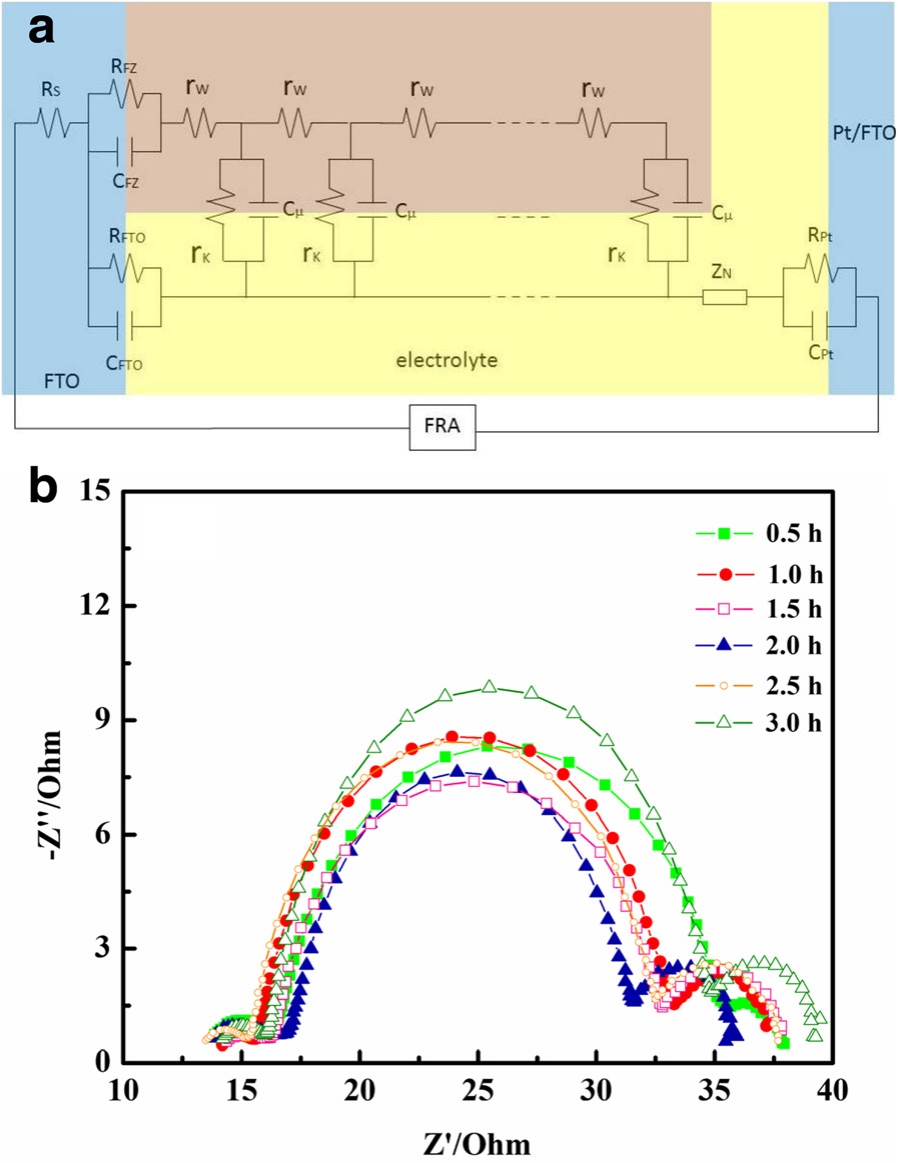
**Equivalent circuit and Nyquist plots.** (**a**) Equivalent circuit for the simulation of impedance spectra. (**b**) Nyquist plots of cells based on 26-μm films. The experimental impedance data were determined under 1 sun AM 1.5 G simulated light.

To determine parameters related to electron transport and recombination, this study used EIS to analyze cells based on 26-μm-thick films. The experimental impedance data, given by the Nyquist plots in Figure 6b, were fitted to an equivalent circuit based on the diffusion-recombination model [42-44] (Figure 6a). The circuit elements related to the ZnO photoelectrode include the electron transport resistance within the ZnO mesoporous film (*R*_w_) (*R*_w_ = *r*_w_*L*, where *L* = film thickness), the charge transfer resistance (*R*_k_) (*R*_k_ = *r*_k_/*L*), which is related to the recombination of electrons at the ZnO/electrolyte interface, and the chemical capacitance of the ZnO electrode (*C*_μ_) (*C*_μ_ = c_μ_*L*). Additional circuit elements were introduced to modify the equivalent circuit model, as described in the following. The series resistance (*R*_S_) represents total transport resistance of the FTO substrates and external circuits. *Z*_N_ is the impedance of the diffusion of I_3_^−^ in the electrolyte. *R*_Pt_ and *C*_Pt_ are the resistance and the capacitance at the Pt/electrolyte interface, respectively. *R*_FTO_ and *C*_FTO_ are the resistance and the capacitance at the FTO/electrolyte interface, respectively. *R*_FZ_ and *C*_FZ_ represent the resistance and the capacitance at the FTO/ZnO interface, respectively. The three fitted parameters of *R*_w_, *R*_k_, and *C*_μ_ can be used to calculate additional parameters, such as the mean electron lifetime (*τ*_eff_), effective electron diffusion coefficient (*D*_eff_), and effective electron diffusion length (*L*_eff_), which are useful for evaluating cell performance.

The Nyquist plots in Figure 6b show the experimental impedance data obtained at various dye adsorption times. The impedance spectra of DSSCs generally exhibit three semicircles. The semicircle in the high-frequency range corresponds to charge transfer behavior at the Pt/electrolyte (*R*_Pt_ and *C*_Pt_), the FTO/electrolyte (*R*_FTO_ and *C*_FTO_), and the FTO/ZnO (*R*_FZ_ and *C*_FZ_) interfaces. The semicircle in the mid-frequency range (the central arc) is assigned to the electron transfer at the ZnO/dye/electrolyte interfaces, which is related to *R*_w_, *R*_k_, and *C*_μ_. The semicircle in the low-frequency range represents the Warburg diffusion process of I^−^/I_3_^−^ in the electrolyte (*Z*_N_) [42-45].

Table 2 presents a summary of results from fitting the experimental impedance data to the equivalent circuit. The highest *R*_k_/*R*_w_ value occurs at a dye adsorption time of 2 h, which is the optimal dye adsorption time for 26-μm-thick photoanodes. Both *J*_SC_ and conversion efficiency reach their maxima at this dye adsorption time (Figure 5). The high *R*_k_/*R*_w_ value obtained at the optimal dye adsorption time suggests that a large number of electrons are injected into the photoelectrode [45,46]. The injected electrons undergo forward transport in the photoanode or recombine with I_3_^−^. This result explains the high *J*_SC_ value observed at the optimal dye adsorption time. In addition, the *k*_eff_ value can be estimated from the characteristic frequency at the top of the central arc (*k*_eff_ = *ω*_max_) of the impedance spectra. The parameter *τ*_eff_ was then estimated as the reciprocal of *k*_eff_ (*τ*_eff_ = 1/*k*_eff_) [45]. Table 2 shows that *τ*_eff_ reaches its highest value at a dye adsorption time of 2 h. Lower *τ*_eff_ values result at insufficient (<2 h) or prolonged dye adsorption times (>2 h). The trend observed here is unlike that of TiO_2_-based cells, whose photovoltaic performance and corresponding EIS spectra remain unchanged after an adsorption time of 12 h [34]. The resistance reaches a constant level once sufficient dye molecules are adsorbed onto the TiO_2_ surfaces, and does not increase at prolonged adsorption times. When the dye adsorption time is insufficient, the ZnO surface is not completely covered with the dye molecules, and certain areas are in direct contact with the electrolyte. Consequently, severe charge recombinations lead to low *τ*_eff_ and *V*_OC_ values. Prolonged dye adsorption times can lead to ZnO dissolution and the formation of Zn^2+^/dye aggregates with acidic dyes [32,35-37], such as the N719 dye used in this study. Dye aggregation leads to slower electron injection and higher charge recombination [36,37]. The end result is a lower *J*_SC_ and overall conversion efficiency [39]. These reports support the trends of *τ*_eff_ and *J*_SC_ versus dye adsorption time observed in this study.

**Table 2:** Effects of dye adsorption time on electron transport properties of fabricated cells

**Dye adsorption time** (**h**)	***R***_**k**_/***R***_**w**_	**Mean electron lifetime** (**ms**)	**Effective electron diffusion time** (**ms**)	**Charge collection efficiency** (%)	**Effective electron diffusion coefficient** (×**10**^−**3**^ **cm**^**2**^ **s**^−**1**^)	**Effective electron diffusion length** (μ**m**)
0.5	5.22	8.40	1.61	80.8	4.21	59.4
1	10.61	12.63	1.19	90.6	5.68	84.7
1.5	13.10	12.63	0.96	92.4	7.01	94.1
2	18.43	15.48	0.84	94.6	8.05	111.6
2.5	10.95	13.91	1.27	90.9	5.86	86.0
3	8.68	12.63	1.46	88.5	3.79	76.6

The thickness of the photoelectrode was 26 μm. *R*_k_, charge transfer resistance at the ZnO/electrolyte interface; *R*_w_, electron transport resistance in the ZnO network.

The effective electron diffusion time (*τ*_d_) in the photoanodes is given by *τ*_d_ = *τ*_eff_/(*R*_k_/*R*_w_). The lowest *τ*_d_ also occurs at the optimal dye adsorption time of 2 h, indicating that the optimal dye adsorption time enhanced electron transport in the ZnO photoanode. Charge collection efficiencies (*η*_CC_) were estimated using the relation *η*_CC_ = 1 − *τ*_d_/*τ*_eff_[47]. Again, *η*_CC_ reaches its maximum value at the optimal dye adsorption time of 2 h, suggesting that using an appropriate dye adsorption time minimizes charge recombination.

The parameter *D*_eff_ was then calculated using the relation *D*_eff_ = (*R*_k_/*R*_w_)(*L*^2^/*τ*_eff_), where *L* is the thickness of the ZnO film (26 μm). The highest *D*_eff_ value (8.05 × 10^−3^ cm^2^ s^−1^) was also obtained at the optimal dye adsorption time of 2 h. This high *D*_eff_ value can be explained by more injected electrons and induced faster transport of electrons. The parameter *L*_eff_, calculated by the relation *L*_eff_ = (*D*_eff_ × *τ*_eff_)^1/2^, reflects the competition between the collection and recombination of electrons. A cell fabricated using the optimal dye adsorption time of 2 h achieved the highest *L*_eff_ value of 111.6 μm, which exceeds the thickness of the photoelectrode (26 μm). This indicates that most of the injected electrons reached the FTO substrate before recombination occurred. This *L*_eff_ trend shows good agreement with that of *J*_SC_. Increased recombination can explain the significant drop in *J*_SC_ values at other dye adsorption times. Overall, the EIS analysis results are in good agreement with the measured device performance parameters.

**Figure 7:**
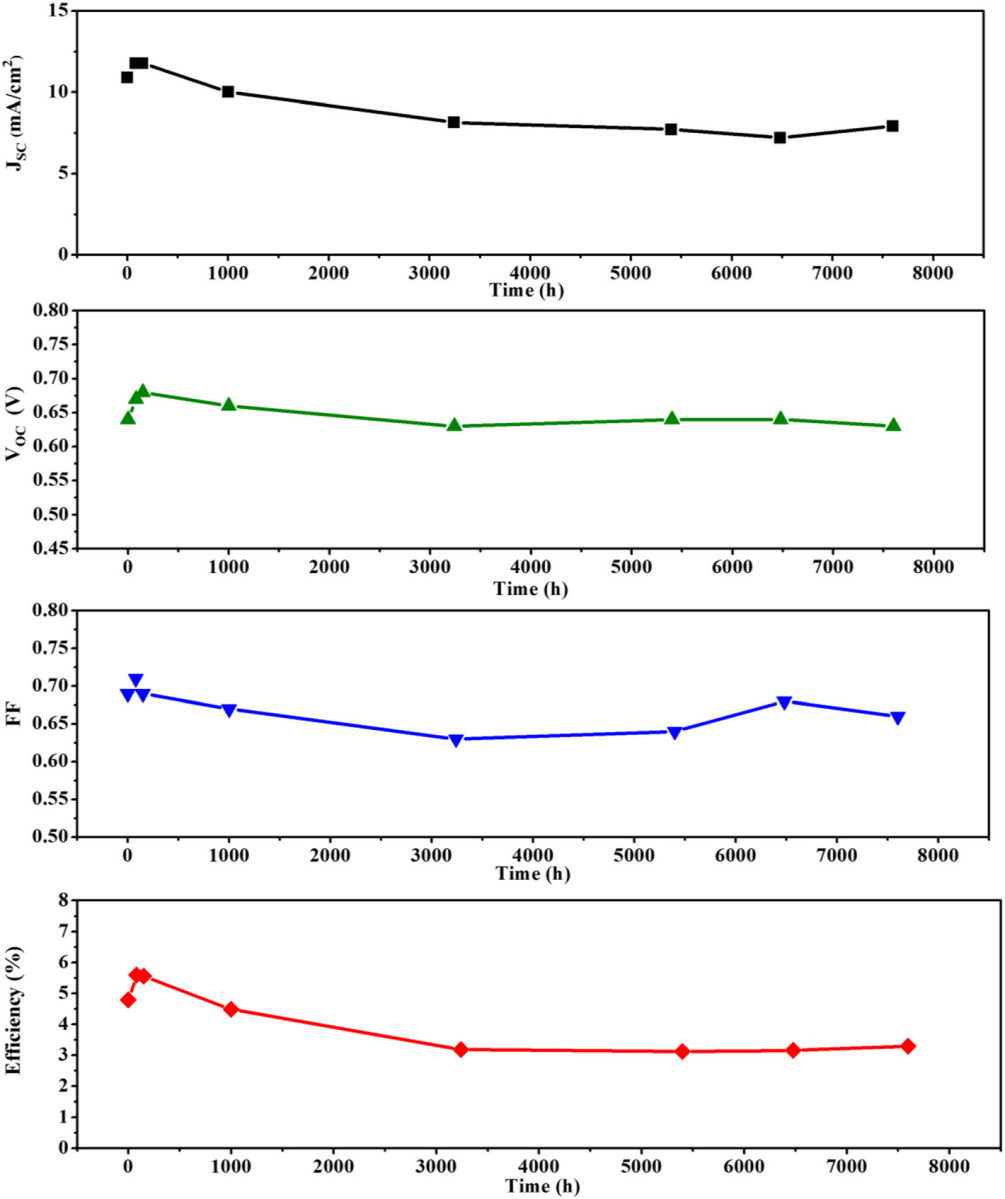
**At**-**rest stability of the best**-**performing cell.** The cell was prepared with a 26-μm film sensitized in a dye solution for 2 h.

The DSSC prepared using the optimized fabrication condition (film thickness = 26 μm and dye adsorption time = 2 h) was also subjected to a long-term at-rest stability test, in which the cell was stored in the dark at room temperature. Figure 7 shows the changes in photovoltaic characteristics over time. The efficiency data shown in this figure are the average of three measurements. During the first 100 h, the device performance improved slightly. The power conversion efficiency increased from 4.76% to 5.61%, whereas *J*_SC_ rose from 10.9 to 11.78 mA/cm^2^. From 100 to 3000 h, the overall conversion efficiency gradually decreased to 3.39% because of the decline of *J*_SC_, *V*_OC_, and FF. Thereafter, the overall conversion efficiency remained nearly unchanged for 8,000 h, as did the *J*_SC_, *V*_OC_, and FF values. Although the fabricated cell used a liquid electrolyte, it demonstrated excellent at-rest stability and retained approximately 70% of its initial efficiency after more than 1 year of storage.

## Conclusions

In summary, this study reports the successful fabrication of DSSC photoelectrodes using commercially available ZnO particles sensitized with acidic N719 dye. The effects of two fabrication factors, the film thickness and the dye adsorption time, were systematically investigated. The results show that to obtain efficient ZnO/N719-based DSSCs, the dye adsorption time must be varied with the photoanode thickness. This is because the dye adsorption time suited for a particular film thickness does not apply to other film thicknesses. This is primarily because prolonged dye sensitization times lead to significant deterioration in the performance of ZnO-based cells. This is in contrast to the typical behavior for TiO_2_-based cells, which usually adopt a single sufficiently long dye adsorption time for all film thicknesses. This is because TiO_2_-based cells are generally insensitive to prolonged sensitization times because of the higher chemical stability of TiO_2_. Through systematic optimization of the film thickness and the dye adsorption time, the highest overall conversion efficiency achieved in this study was 5.61%, obtained from a 26-μm photoelectrode sensitized for 2 h. The best-performing cell also showed remarkable at-rest stability, retaining approximately 70% of its initial efficiency after more than 1 year of room-temperature storage in the dark.

## Abbreviations

AM: Air mass; DSSC: Dye-sensitized solar cells; EIS: Electrochemical impedance spectroscopy; FE-SEM: Field-emission scanning electron microscopy; FF: Fill factor; FTO: Fluorine-doped tin oxide; *J*_SC_: Short-circuit photocurrent density; *V*_OC_: Open circuit voltage; XRD: X-ray diffraction.

## Competing interests

The authors declare that they have no competing interests.

## Authors’ contributions

WCC designed and performed the experiment, analyzed the data, and helped draft the manuscript. CML helped draft the manuscript. WCY conceived the study, participated in its design and coordination, and helped with the manuscript preparation. CHL helped draft the manuscript. All authors read and approved the final manuscript.

## References

[B1] Nazeeruddin MK De Angelis F Fantacci S Selloni A Viscardi G Liska P Ito S Takeru B Grätzel MG Combined experimental and DFT-TDDFT computational study of photoelectrochemical cell ruthenium sensitizers J Am Chem Soc 2005 127 16835 16847 10.1021/ja052467l 16316230

[B2] Chen CY Wang MK Li JY Pootrakulchote N Alibabaei L Ngoc-Le CH Decoppet JD Tsai JH Grätzel C Wu CG Zakeeruddin SM Grätzel M Highly efficient light-harvesting ruthenium sensitizer for thin-film dye-sensitized solar cells ACS Nano 2009 3 3103 3109 10.1021/nn900756s 19746929

[B3] Hara K Horiguchi T Kinoshita T Sayama K Sugihara H Arakawa H Highly efficient photon-to-electron conversion with mercurochrome-sensitized nanoporous oxide semiconductor solar cells Sol Energy Mater Sol Cells 2000 64 115 134 10.1016/S0927-0248(00)00065-9

[B4] Sayama K Sugihara H Arakawa H Photoelectrochemical properties of a porous Nb2O5 electrode sensitized by a ruthenium dye Chem Mater 1998 10 3825 3832 10.1021/cm980111l

[B5] Katoh R Furube A Yoshihara T Hara K Fujihashi G Takano S Murata S Arakawa H Tachiya M Efficiencies of electron injection from excited N3 into nanocrystalline semiconductor (ZrO2, TiO2, ZnO, Nb2O5, SnO2, In2O3) films J Phys Chem B 2004 108 4818 4822 10.1021/jp031260g

[B6] Quintana M Edvinsson T Hagfeldt A Boschloo G Comparison of dye-sensitized ZnO and TiO2 solar cells: studies of charge transport and carrier lifetime J Phys Chem C 2007 111 1035 1041 10.1021/jp065948f

[B7] Gao YF Nagai M Chang TC Shyue JJ Solution-derived ZnO nanowire array film as photoelectrode in dye-sensitized solar cells Cryst Growth Des 2007 7 2467 2471 10.1021/cg060934k

[B8] Jiang CY Sun XW Lo GQ Kwong DL Wang JX Improved dye-sensitized solar cells with a ZnO-nanoflower photoanode Appl Phys Lett 2007 90 26 263501 10.1063/1.2751588

[B9] Hosono E Fujihara S Honna I Zhou H The fabrication of an upright-standing zinc oxide nanosheet for use in dye-sensitized solar cells Adv Mater 2005 17 2091 2094 10.1002/adma.200500275

[B10] Zhang Q Dandeneau CS Zhou X Cao G ZnO nanostructures for dye-sensitized solar cells Adv Mater 2009 21 4087 4108 10.1002/adma.200803827

[B11] Zhang Q Cao G Nanostructured photoelectrodes for dye-sensitized solar cells Nano Today 2011 6 91 109 10.1016/j.nantod.2010.12.007

[B12] Martinson ABF Elam JW Hupp JT Pellin MJ ZnO nanotube based dye-sensitized solar cells Nano Lett 2007 7 2183 2187 10.1021/nl070160+ 17602535

[B13] Zhang Q Myers D Lan J Jenekhe SA Applications of light scattering in dye-sensitized solar cells Phys Chem Chem Phys 2012 14 14982 14998 23042288 10.1039/c2cp43089d

[B14] Wang ZS Kawauchi H Kashima T Arakawa H Significant influence of TiO2 photoelectrode morphology on the energy conversion efficiency of N719 dye-sensitized solar cell Coord Chem Rev 2004 248 1381 1389 10.1016/j.ccr.2004.03.006

[B15] Kang SH Kim JY Kim HS Koh HD Lee JS Sung YE Influence of light scattering particles in the TiO2 photoelectrode for solid-state dye-sensitized solar cell J Photochem Photobiol A 2008 200 294 300 10.1016/j.jphotochem.2008.08.010

[B16] Ito S Nazeeruddin M Liska P Comte P Charvet R Péchy P Jirousek M Kay A Zakeeruddin S Grätzel M Photovoltaic characterization of dye-sensitized solar cells: effect of device masking on conversion efficiency Prog Photovolt Res Appl 2006 14 589 601 10.1002/pip.683

[B17] Hore S Vetter C Kern R Smit H Hinsch A Influence of scattering layers on efficiency of dye-sensitized solar cells Sol Energy Mater Sol Cells 2006 90 1176 1188 10.1016/j.solmat.2005.07.002

[B18] Ito S Nazeeruddin M Zakeeruddin S Péchy P Comte P Grätzel M Mizuno T Tanaka A Koyanagi T Study of dye-sensitized solar cells by scanning electron micrograph observation and thickness optimization of porous TiO2 electrodes Int J Photoenergy 2009 2009 517609

[B19] Ito S Murakami T Comte P Liska P Grätzel C Nazeeruddin M Grätzel M Fabrication of thin film dye sensitized solar cells with solar to electric power conversion efficiency over 10% Thin Solid Films 2008 516 4613 4619 10.1016/j.tsf.2007.05.090

[B20] Qiu Y Chen W Yang S Double-layered photoanodes from variable-size anatase TiO2 nanospindles: a candidate for high-efficiency dye-sensitized solar cells Angew Chem Int Ed 2010 49 3675 3679 10.1002/anie.200906933 20376867

[B21] Tan B Wu YY Dye-sensitized solar cells based on anatase TiO2 nanoparticle/nanowire composites J Phys Chem B 2006 110 15932 15938 10.1021/jp063972n 16898747

[B22] Kevin M Fou YH Wong ASW Ho GW A novel maskless approach towards aligned, density modulated and multi-junction ZnO nanowires for enhanced surface area and light trapping solar cells Nanotechnology 2010 21 315602 315610 10.1088/0957-4484/21/31/315602 20634568

[B23] Tetreault N Horvath E Moehl T Brillet J Smajda R Bungener S Cai N Wang P Zakeeruddin SM Forro L Magrez A Grätzel M High-efficiency solid-state dye-sensitized solar cells: fast charge extraction through self-assembled 3D fibrous network of crystalline TiO2 nanowires ACS Nano 2010 4 7644 7650 10.1021/nn1024434 21082857

[B24] Lin CJ Yu WY Chien SH Effect of anodic TiO2 powder as additive on electron transport properties in nanocrystalline TiO2 dye-sensitized solar cells Appl Phys Lett 2007 91 233120 10.1063/1.2823604

[B25] Nakayama K Kubo T Nishikitani Y Deposited TiO2 nanotube light-scattering layers of electrophoretically dye-sensitized solar cells Jpn J Appl Phys 2008 47 6610 6614 10.1143/JJAP.47.6610

[B26] Chou TP Zhang QF Fryxell GE Cao GZ Hierarchically structured ZnO film for dye-sensitized solar cells with enhanced energy conversion efficiency Adv Mater 2007 19 2588 2592 10.1002/adma.200602927

[B27] Zhang Q Chou TP Russo B Jenekhe SA Cao G Polydisperse aggregates of ZnO nanocrystallites: a method for energy-conversion-efficiency enhancement in dye-sensitized solar cells Adv Funct Mater 2008 18 1654 1660 10.1002/adfm.200701073

[B28] Yan K Qiu Y Chen W Zhang M Yang S A double layered photoanode made of highly crystalline TiO2 nanooctahedra and agglutinated mesoporous TiO2 microspheres for high efficiency dye sensitized solar cells Energy Environ Sci 2011 4 2168 2176 10.1039/c1ee01071a

[B29] Zhang Q Park K Xi J Myers D Cao G Recent progress in dye-sensitized solar cells using nanocrystallite aggregates Adv Energy Mater 2011 1 988 1001 10.1002/aenm.201100352

[B30] Lee B Hwang DK Guo P Ho ST Buchholtz DB Wang CY Chang RPH Materials, interfaces, and photon confinement in dye-sensitized solar cells J Phys Chem B 2010 114 14582 14591 10.1021/jp102359r 21070056

[B31] Hsu CP Lee KM Huang JTW Lin CY Lee CH Wang LP Tsai SY Ho KC EIS analysis on low temperature fabrication of TiO2 porous films for dye-sensitized solar cells Electrochim Acta 2008 53 7514 7522 10.1016/j.electacta.2008.01.104

[B32] Chou TP Zhang QF Cao GZ Effects of dye loading conditions on the energy conversion efficiency of ZnO and TiO2 dye-sensitized solar cells J Phys Chem C 2007 111 18804 18811 10.1021/jp076724f

[B33] Lee KM Suryanarayanan V Huang JH Justin Thomas KR Lin JT Ho KC Enhancing the performance of dye-sensitized solar cells based on an organic dye by incorporating TiO2 nanotube in a TiO2 nanoparticle film Electrochim Acta 2009 54 4123 4130 10.1016/j.electacta.2009.02.052

[B34] Kim JK Seo H Son MK Shin I Hong J Kim HJ The analysis of the change in the performance and impedance of dye-sensitized solar cell according to the dye-adsorption time Curr Appl Phys 2010 10 S418 S421 10.1016/j.cap.2010.02.024

[B35] Horiuchi H Katoh R Hara K Yanagida M Murata S Arakawa H Tachiya M Electron injection efficiency from excited N3 into nanocrystalline ZnO films: effect of (N3-Zn2+) aggregate formation J Phys Chem B 2003 107 2570 2574

[B36] Keis K Lindgren J Lindquist SE Hagfeldt A Studies of the adsorption process of Ru complexes in nanoporous ZnO electrodes Langmuir 2000 16 4688 4694 10.1021/la9912702

[B37] Qin Z Huang YH Qi JJ Qu L Zhang Y Improvement of the performance and stability of the ZnO nanoparticulate film electrode by surface modification for dye-sensitized solar cells Colloids Surf A 2011 386 179 184 10.1016/j.colsurfa.2011.07.011

[B38] Sakuragi Y Wang XF Miura H Matsui M Yoshida T Aggregation of indoline dyes as sensitizers for ZnO solar cells J Photochem Photobiol A 2010 216 1 7 10.1016/j.jphotochem.2010.08.015

[B39] Yan FP Huang LH Zheng JS Huang J Lin Z Huang F Wei MD Effect of surface etching on the efficiency of ZnO-based dye-sensitized solar cells Langmuir 2010 26 7153 7156 10.1021/la904238n 20112927

[B40] Thavasi V Renugopalakrishnan V Jose R Ramakrishna S Controlled electron injection and transport at materials interfaces in dye sensitized solar cells Mater Sci Eng R 2009 63 81 99 10.1016/j.mser.2008.09.001

[B41] Saito M Fujihara S Large photocurrent generation in dye-sensitized ZnO solar cells Energy Environ Sci 2008 1 280 283 10.1039/b806096g

[B42] Juan B Theory of the impedance of electron diffusion and recombination in a thin layer J Phys Chem B 2002 106 325 333 10.1021/jp011941g

[B43] Wang KP Teng H Zinc-doping in TiO2 films to enhance electron transport in dye-sensitized solar cells under low-intensity illumination Phys Chem Chem Phys 2009 11 9489 9496 19830333 10.1039/b912672d

[B44] Chang WC Cheng YY Yu WC Yao YC Lee CH Ko HH Enhancing performance of ZnO dye-sensitized solar cells by incorporation of multiwalled carbon nanotubes Nanoscale Res Lett 2012 7 166 172 10.1186/1556-276X-7-166 22390565 PMC3320520

[B45] Adachi M Sakamoto M Jiu J Ogata Y Isoda S Determination of parameters of electron transport in dye-sensitized solar cells using electrochemical impedance spectroscopy J Phys Chem B 2006 110 13872 13880 10.1021/jp061693u 16836336

[B46] Lee CH Chiu WH Lee KM Yen WH Lin HF Hsieh WF Wu JM The influence of tetrapod-like ZnO morphology and electrolytes on energy conversion efficiency of dye-sensitized solar cells Electrochim Acta 2010 55 8422 8429 10.1016/j.electacta.2010.07.061

[B47] Wang Q Zhang Z Zakeeruddin SM Grätzel M Enhancement of the performance of dye-sensitized solar cell by formation of shallow transport levels under visible light illumination J Phys Chem C 2008 112 7084 7092 10.1021/jp800426y

